# Supplementary material for: Long-term neurological consequences following benzodiazepine exposure: A scoping review
Source: PLoS One. 2025 Aug 28;20(8):e0330277. doi: 10.1371/journal.pone.0330277 (PMC12393751; doi:10.1371/journal.pone.0330277)
Supplement: Attachment — Submitted filename: Combined response to reviewers.docx [file pone.0330277.s002.docx]

PONE-D-25-04510

Long-Term Neurological Consequences Following Benzodiazepine Exposure: A Scoping Review.

PLOS ONE

Dear Editors:

This Response to Reviewers document contains all three responses to reviewers. The first two have previously been provided to PLoS One, and are included here for completeness.

1. First response, dated 2/26/2025, in response to the initial review at PLoS One provided to me on 11/22/24.
2. Second response, dated 6/17/2025, in response to the PLoS One review emailed to me on 6/5/2025.
3. Third response, dated 7/22/2025, in response to the PLoS One request emailed to me on 6/22/2025.

**First Response**

February 26, 2025

**Alexis Ritvo, MD MPH**

Department of Psychiatry

1890 N Revere Ct, Suite 4020 | Aurora, CO 80045

**o** 303 724 9416 | **f** 303 724 9472 | Alexis.Ritvo@cuanschutz.edu

medschool.cuanschutz.edu/psychiatry

Dear Editors,

On behalf of the authors, it is my pleasure to re-submit to you “Long-Term Neurological Consequences Following Benzodiazepine Exposure: A Scoping Review.” The long-term neurological consequences of benzodiazepine exposure are much less familiar than the symptoms and risks of acute withdrawal. A scoping review was conducted of electronic databases for studies that reported on patient outcomes four or more weeks after complete cessation of benzodiazepine use.

We are very appreciative of our initial review at PLOS ONE on 11/22/24, which asked us to update our literature search originally completed August 2023 to encompass the most recent publications. As your reviewers suggested, we are submitting this updated manuscript again for consideration. Below we provide a response to review comments about requested changes.

In total in this study, forty-six results were retrieved, some of which provided signals for protracted symptoms, often reported as incidental findings, and others that showed benzodiazepine discontinuation was beneficial. The results suggest that the benefits of benzodiazepine discontinuation for many patients tended to obscure the more prolonged, severe, and sometimes debilitating symptoms that persisted for months and years in a subpopulation of patients.

All authors contributed equally to this manuscript, albeit from different perspectives. This is an original article and is not under consideration or submitted elsewhere nor have these results been reported elsewhere. We appreciate your consideration and look forward to hearing from you.

Sincerely,

Alexis Ritvo, MD MPH (On behalf of the authors)

Assistant Professor of Psychiatry

Program Director, Addiction Psychiatry Fellowship

**Comment 1:** We are concerned that the literature review was completed in 09 August 2023. Systematic and scoping reviews should provide an up-to-date overview of the research topic covered. Since the literature search was completed over a year ago, we are concerned that this study does not meet this requirement.

**Author’s response 1:** We conducted a bridge search in December 2024 and amended the manuscript accordingly. Although the text changes to our manuscript were minimal (lines 205-210 only in Methods section), we conducted a bridge search which retrieved an additional 539 articles. These articles were then reviewed by the authors. In the end, we found no additional articles to include in our study, and none of our tables or content were affected. However, this was a substantial effort to make sure we included all of the latest literature. Figure 1 (PRISMA chart) lines 249-251 was correspondingly updated to reflect the supplementary search.

**Comment 2:** We kindly request you to clearly outline the Inclusion / Exclusion criteria in the revised version of the manuscript.

**Author’s response 2:** Our inclusion/exclusion criteria are outlined in the manuscript in lines 189-214. Additionally, Appendix 1 provides the detailed research protocol and Appendix 2 provides the librarian search strategies.

**Second Response (dated 6/17/2025)**

Thank you for the peer review comments, which we have addressed below in the table.

|  | Questions | Reviewer’s comment | Authors’ input | Action(s) taken |
| --- | --- | --- | --- | --- |
| **1** | **Is the manuscript technically sound, and do the data support the conclusions?** | Partly | This is a scoping review and meets the standards for such. Without more specifics, it is difficult for us to address this. | None |
| 2 | **Has the statistical analysis been performed appropriately and rigorously?** | N/A | Agreed | None |
| 3 | **Have the authors made all data underlying the findings in their manuscript fully available?** | No | This comment confuses us. We have supplied a detailed search strategy (databases, flowchart, keyword lists) and inclusion/exclusion criteria. We feel that our research could be duplicated by any who followed the methodology we disclosed. We do not know what other underlying data are required | None |
| 4 | **Is the manuscript presented in an intelligible fashion and written in standard English?** | Yes | Thank you | None |
| 5A | Reviewer comments invited | The authors should clarify the rationale for defining a two- to four-week period as the threshold for benzodiazepine exposure risk. | We established the study criteria as patients ≥18 years who had taken BZD chronically, long terms, or consistently over a period of > 2 wk and who had discontinued them for >4 weeks. This is in line with the published literature and the literature review recently conducted for the ASAM BZD tapering guidelines. This guidance states that physical dependence may occur in as little as 2 wk and symptoms of protracted withdrawal may emerge as early as 4 wk post-discontinuation. | We originally cited the FDA boxed warning for this (original reference #84). We  substituted the ASAM guidelines (Brunner 2025), which makes the statements noted in the column to the left and also references the FDA boxed warning. We identified 8 additional references used to support this statement, and have included only the 5 with the highest quality evidence.  If more references are desired, we will gladly add the other three: . Maine BZD study group 2008, Pottie 2018, and Hood 2014. |
| 5B |  | As various benzodiazepines were included, and the reviewed studies do not consistently examine the same agents, the authors are encouraged to discuss both the differences and any potential uniformities across these drugs. | This was a scoping review looking for evidence of protracted withdrawal within a class of medications. This is a common approach in this sort of research. A good example of this approach is NIH Project Number5R01AG060975 which treats benzodiazepines as a class.  Since this is the first such study of its kind, the authors felt that it was wise to approach it in broad strokes. Fine-tuning it to specific agents exceeds our original goals. This would be a worthwhile topic for future research or a secondary analysis. However, as we started the project, it was unclear what, if anything, we would find. | None |
| 5C |  | Furthermore, the duration of use, the ratio of duration to discontinuation, and the timing of discontinuation should be more clearly addressed in relation to the development of withdrawal symptoms. | The withdrawal symptoms in our study are not described by the studies in any in-depth way given the broad nature of studies accepted by our scoping review. We reported mainly on signals. The data as to duration of use, etc. to which we could reliably anchor these findings is spotty and inconsistent between the studies. None of the studies were looking for withdrawal symptoms but mentioned them as incidental findings.  The interest in associating duration of use, etc. to our findings might introduce what could be potential bias to our scoping review. Our purpose was to look for the presence of withdrawal symptoms and not adjudicate their causes.  By only looking for specific things (e.g., withdrawal related to duration of use), we defeat the purpose of the scoping review, which was just to look for evidence for withdrawal symptoms after benzodiazepine discontinuation. | None |
| 6 | **PLOS authors have the option to publish the peer review history of their article. If published, this**  **will include your full peer review and any attached files.** | N/A | No | None |

In conclusion, we appreciate the comments offered to us, in particular suggestions that would be very worthwhile subjects for secondary analysis or future endeavors. Our scoping review is a first step in these efforts, and we hope not the last in our investigation into this subject.

We acknowledge the previously noted competing interests. This does not alter our adherence to PLOS ONE policies on sharing data and materials.

On behalf of the authors, I remain,

Yours truly,

Alexis Ritvo, MD

**Third Response (dated 7/22/2025)**

7/22/25

RE: PONE-D-25-04510R1
Long-term neurological consequences following benzodiazepine exposure: A scoping review.

thread::5pqOJXNWduGpg85GbvLJRxo::

Dear Editors:

Thank you for the notification provided via email on June 22, 2025, requesting that we

“Please confirm at this time whether or not your submission contains all raw data required to replicate the results of your study.”

We have reviewed the manuscript and its supplemental material. In the prior submission, the values used to build the graph in figure 3 were provided in tables 1, 2 and 3, and no other means, standard deviations and other measures were reported.

The Study Protocol, Search Strategies and PRISMA Updated Checklist were provided previously as Appendices 1-3, respectively. We are submitting an additional Appendix 4, “Excluded studies and the reasons for their exclusion”.^1^ Appendices 1-4 will allow easy replication of the study.

None of the other files were modified from the prior submittal, but we are resubmitting the manuscript and its marked-up version per your requirements.

We hope that this meets the requirements, and look forward to seeing this article published.

Sincerely,

Alexis D. Ritvo, MD

Note 1:

We are providing this additional information because at least two prior PLoS One publications provided the same type of information:

Haykal KA, Pereira L, Power A, Fournier K. Medical student wellness assessment beyond anxiety and depression: A scoping review. *PLoS One*. 2022;17(10):e0276894. Published 2022 Oct 27. doi:10.1371/journal.pone.0276894

McWilliams S, Singh I, Leung W, Stockler S, Ipsiroglu OS. Iron deficiency and common neurodevelopmental disorders-A scoping review. *PLoS One*. 2022;17(9):e0273819. Published 2022 Sep 29. doi:10.1371/journal.pone.0273819

<https://journals.plos.org/plosone/article?id=10.1371/journal.pone.0273819#sec025> —> select "Supporting information"
